# Effectiveness of computerized point-of-care reminders on adherence with multiple clinical recommendations by primary health care providers: protocol for a cluster-randomized controlled trial

**DOI:** 10.1186/s40064-016-3124-2

**Published:** 2016-09-07

**Authors:** Leonardo Méndez Boo, Ermengol Coma, Manuel Medina, Eduardo Hermosilla, Manuel Iglesias, Carmen Olmos, Sebastian Calero Muñoz, Johanna Caro Mendivelso

**Affiliations:** 1 Sistemes d’Informació d’Atenció Primària (SISAP), Institut Català de la Salut, Barcelona, Catalonia Spain; 2 Institut Universitari d’Investigació en Atenció Primària Jordi Gol (IDIAP Jordi Gol), Gran Via de les Corts Catalanes 587, 08007 Barcelona, Catalonia Spain; 3 Oficina Projecte ECAP, Centre de competència funcional, Institut Català de la Salut, Barcelona, Catalonia Spain; 4 Àrea de Desenvolupament Clínic, Institut Català de la Salut, Barcelona, Spain

**Keywords:** Reminder systems, Quality of health care, Primary health care, Electronic health record

## Abstract

**Background:**

To determine the effectiveness of reminders compared to no reminders in improving adherence to multiple clinical recommendations measured as the resolution of the clinical condition that motivated the reminder, in a primary care setting with a well-established feedback system.

**Methods/design:**

A 12-month, cluster-randomized, controlled clinical trial was designed (randomized by primary care team) to evaluate the impact of computerized reminders. All study participants will continue to receive the usual feedback from the electronic health records system. The control group (well-established feedback) will be compared with reminders and a well-established feedback system. The study will include all general practitioners (3425) and nurses (3262) providing primary care for a population aged 14 years or older in the 282 primary care teams reporting to the Catalan Institute of Health. Up to 10 clinical reminders are offered for each patient, recommending action related to at least one of nine clinical conditions: arterial hypertension, elevated cardiovascular risk, type 2 diabetes mellitus, cerebrovascular accident, ischemic heart disease, heart failure, atrial fibrillation, smoking habit, and hepatitis C. The outcomes are the resolution of the clinical condition that motivated the reminder and the time elapsed between the first reminder message and implementation of the recommended action (months). Due to the obvious correlation between reminders about the same patient, the profile of patients assigned to a particular professional, and the professionals assigned to a particular centre, hierarchical modelling will be used to simultaneously estimate the effect of the study variables at these different levels of analysis. To estimate the impact of the intervention arm, an analysis of adherence to each type of reminder will be carried out, using multi-level logistical regression models at level of the primary care centre. Time to adherence will be estimated by the Kaplan–Meier method and comparisons will be done using the log-rank test.

**Discussion:**

The results of this study could provide new evidence on the impact of computerized reminders at the point of care on adherence to clinical guidelines in primary care with an established feedback system.

*Trial registration* ISRCTN42391639. October 8, 2012

## Background

Computerization of medical records and the availability of computer access in all medical offices in the primary care setting have made it possible to study the impact of this computerization in daily clinical practice (Fina et al. 2006). The Catalan Institute of Health (*Institut Català de la Salut*, ICS) is the main public provider of primary care services in the National Health System of Catalonia, with 282 primary care teams serving nearly 6 million people (80 % of the Catalan population). The 8000 health professionals comprising these teams provide family, paediatric, and nursing care. Since 2005, all ICS primary care providers have used the same electronic health records system, called ECAP, and since 2006 have been able to access a feedback system, updated monthly, that reports on quality indicators evaluating the care provided. All professionals have access to their rates of adherence to the evaluation standards and can view detailed information at the patient level. The feedback system also compiles a list of patients who do not meet all established criteria of the 62 evidence-based recommendations that constitute a unique index known as the Quality of Care Standard (*Estàndard de Qualitat Assistencial*, EQA). At present, health professionals have point-of-care access to all of these results, with no additional data-recording effort on their part (Coma et al. 2013).

Primary health care allows longitudinal follow-up of a population. It also requires attention to multiple health conditions and disease prevention activities in a relatively brief office visit. Bodenheimer estimated that a primary care physician would require 18 h per workday to implement all of the prevention activities recommended by experts in chronic diseases (Bodenheimer 2008). Given the multitude of recommendations, it would be of great interest to health care professionals to have tools available that would help them carry out evidence-based activities. One of the strategies used to improve clinical practice is the incorporation of recommended activities as reminders in the clinical record (Jamtvedt et al. 2003).

The availability of the shared electronic health record allows electronic reminders at the point of care, which has been associated with an improvement in clinical practice, estimated at 4.2 % (interquartile range 0.8–18.8 %). This wide variability could be explained by differences in the settings where the studies were carried out, the basal levels of adherence, or the characteristics of the reminders studied (Shojania et al. 2010).

Most of the randomized clinical trials that have evaluated the effectiveness of reminders in the primary care setting have included reminders for just one disease or clinical condition (Eccles et al. 2002; Filippi et al. 2003; Flottorp et al. 2002; Hicks et al. 2008; Krall et al. 2004; Safran et al. 1995; Sequist et al. 2005; Tierney et al. 2003; van Wyk et al. 2008). The studies that have included interventions with multiple reminders have focussed on preventive measures such as vaccinations and screenings or potential adverse effects of medications (Frank et al. 2004; Tamblyn et al. 2003).

In a retrospective analysis, an association was observed between the level of adherence to the recommendations of interest and the frequency of accessing the feedback provided and the patient list. In other words, the professionals who consulted their own quality results and the status of their patient lists showed greater improvement in their clinical indicators than their colleagues who did not consult the feedback results; in addition, a temporal relationship was detected between the availability of feedback and the clinical improvement (Fina et al. 2006). Even though different clinical trials have studied the impact of paper reminders and other interventions such as economic incentive or educational support materials, there is a lack of scientific evidence on the impact of computerized reminders (Shojania et al. 2010).

In 2012, a maximum of 10 computerized reminders per patient was introduced into the ECAP system, visible to the health professional during the patient visit and related to 25 of the 62 recommendations considered as quality indicators in the EQA. We designed a clinical trial to evaluate whether adding computerized reminders to a well-established feedback system would improve adherence to multiple clinical recommendations in the primary care setting.

### Research hypothesis

The hypothesis was that computerized point-of-care reminders (i.e., presented during the contact between a primary care professional and a patient) improve the level of adherence to the recommended action and reduce the time to implementation of the recommendation.

### Objectives

#### Primary objectives

To determine the effectiveness of reminders compared to no reminders in improving adherence to multiple clinical recommendations measured as the resolution of the clinical condition that motivated the reminder, in a primary care setting with a well-established feedback system.

#### Secondary objectives

To describe the effectiveness of the three levels of reminders intervention: (1) pop-up reminder, (2) pop-up reminder and a calendar icon, and (3) pop-up reminder, calendar icon, and configurability (users can select how the reminders are shown).

To describe resolution and time to resolution of the reminders by type of professional, primary care team and by each of the reminders in improving adherence to the clinical recommendations contained in the reminders.

To compare the effectiveness of computerized reminders in improving adherence to multiple clinical recommendations by category (primary, secondary and tertiary prevention).

To compare the effectiveness of computerized reminders in improving adherence to multiple clinical recommendations by category (professional action only and the professional action plus the modification of patient behaviour).

## Methods

### Study design

The study is a controlled clinical trial, cluster-randomized by primary care team, designed to evaluate the impact of computerized reminders on adherence to clinical recommendations in a primary care setting with an established feedback system. A pilot study will conducted with primary care centre (PCC) health professionals to improve the development of the reminders. During the clinical trial, all of the study participants, including the control group, continued to receive the usual feedback. The control group is compared with each of the three study arms: (1) pop-up reminder, (2) pop-up reminder and a calendar icon, and (3) pop-up reminder, calendar icon, and configurability.

### Study population

The inclusion criteria are: being a family physician or nurse who care for adult patients aged 14 years of older with clinical conditions that could generate at least one of the reminders in one of the 282 primary care teams managed by ICS. The exclusion criteria are: professionals who did not adopt the reformed model of primary care defined in Spanish legislation will be excluded due to low utilization of the electronic health record (Real Decreto 137/1984). Furthermore professionals of a primary care team participating in other clinical trials of electronic health record reminders, and those that participate in the pilot stage of this clinical trial will be excluded. It is important to emphasize that this is a global intervention involving all ICS health professionals. The analysis will take into account all included professionals in each PCC (or cluster).

#### Control group

During the clinical trial, all study participants, including those in the control group, will continue to receive the usual feedback established in 2006. This feedback consists of a monthly update for each family physician and primary care nurse of their percentage of adherence to each of the evidence-based recommendations in the EQA, along with a comparison of the month’s results with their own basal data, with the rest of their team, and with the mean of all ICS teams.

**Fig. 1:**
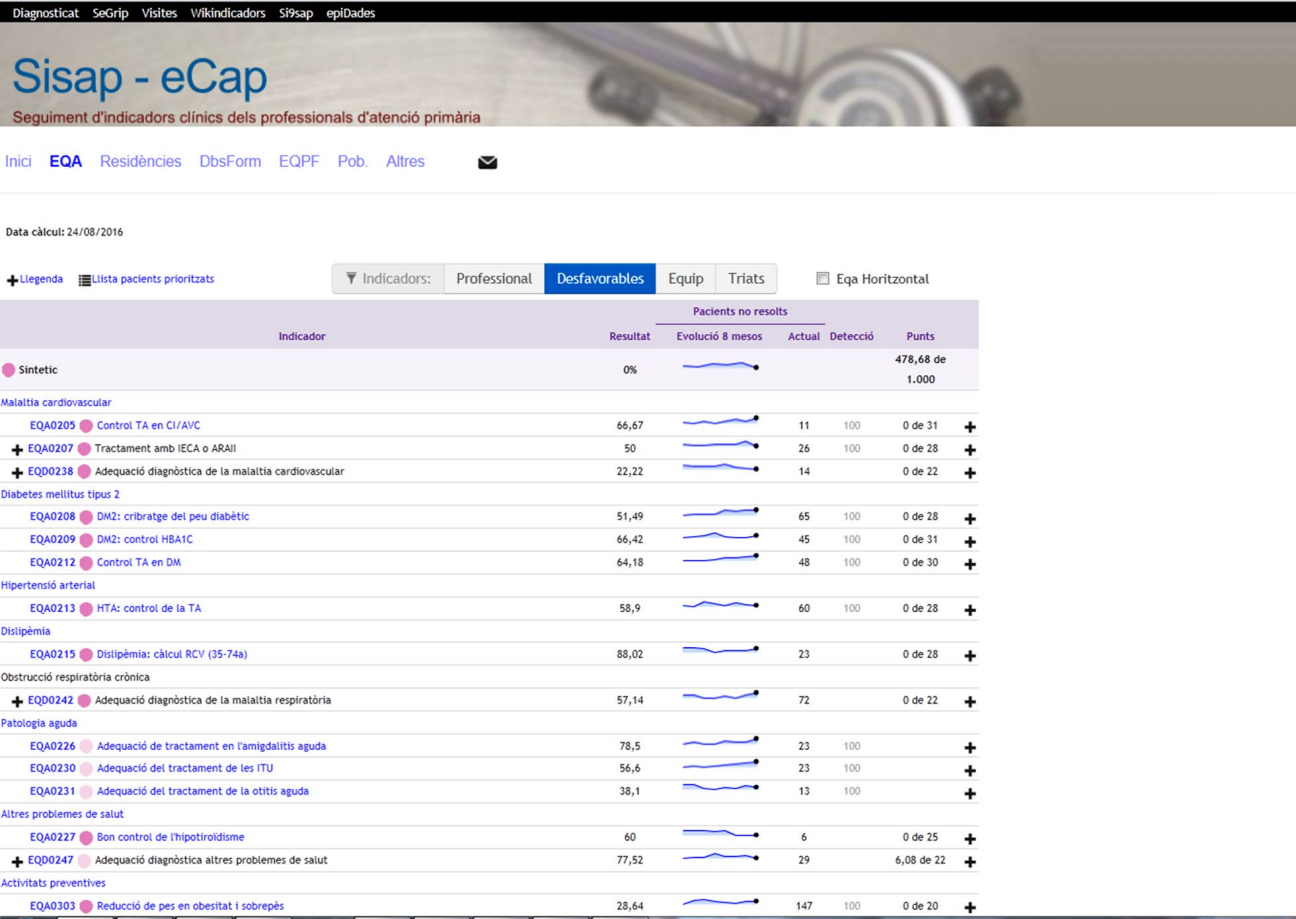
Feedback screen that can be accessed by health professionals from the electronic health records system. Well-established feedback without reminders (control group)

**Fig. 2:**
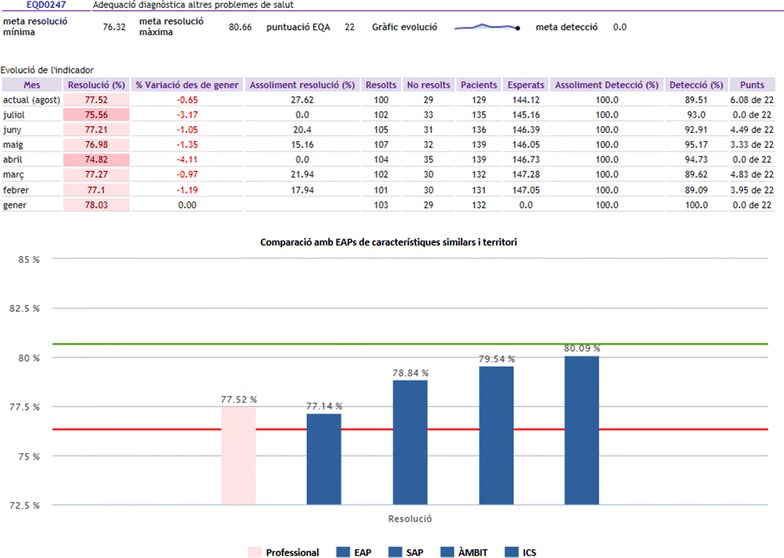
Detail of the feedback screen to which health professionals have access. Well-established feedback without reminders (control group)

For ease of interpretation, the results will be color-coded by target values. For 20 of the 25 recommendations, in addition to the numerical score, professionals will be able to consult the list of patients who do not meet the target; the goal is that the professional will review and take action with those individuals. Access to the feedback screen is provided as a direct link within the electronic health record (Figs. 1, 2, in Catalan; an online demonstration of this feedback system is available at: http://www.amf-semfyc.com/sisap/) (Coma et al. 2013). The monthly results are also used as part of a performance incentive variable in the compensation model; during the intervention year, the incentive will not exceed a total of 750 euros per physician and 380 euros per nurse.

In contrast to the intervention with point-of-care reminders that will be evaluated using the present protocol, the usual monthly feedback screen (control group) does not allow direct interaction with the clinical health record system; this feedback is associated with the professional, not the individual patients, and requires that the health professional actively seek out the numerical results or the patient lists from which the established feedback screen draws the relevant data.

### Intervention

A maximum of 10 clinical reminders per patient will be offered, recommending actions related to at least one of the following nine clinical conditions: arterial hypertension, elevated cardiovascular risk, type 2 diabetes mellitus, cerebrovascular accident, ischemic heart disease, heart failure, atrial fibrillation, smoking habit, and hepatitis C. The reminders are integrated into the electronic health record for each patient so that they are available to any health professional who cares for the patient; the information is updated weekly to reflect any changes that occur.

Each reminder consists of the following information: an orange or red icon that codes the importance of the recommendation as moderate or high, respectively; the relevant clinical condition; the recommended action; and the date and value entered for the recommendation or related laboratory results. A recommendation is classified as highly important if treatment is required (red icon: e.g., atrial fibrillation, anticoagulant/antiplatelet treatment, 12/06/2011) and as moderately important if it refers to a variable, laboratory result, or vaccination (orange icon: e.g., Type 2 diabetes mellitus, monitor HbA1c, most recent value, 8.3, 12/05/2011) (Table 1).

**Table 1:** Recommendations, clinical conditions, importance and estimated number of reminders

Recommendation (http://www.gencat.cat/ics/professionals/guies/mpoc/mpoc.htm)	Clinical condition	Importance	n (estimated)
Control blood pressure: ≤140/90 or ≥150/95, depending on risk status	Arterial hypertension/elevated cardiovascular risk	Moderate	534,702
Control glycaemia: HbA1c ≤ 8 %	Type 2 diabetes mellitus	Moderate	92,520
Control cholesterol: LDL < 120	Cerebrovascular accident/Ischemic heart disease	Moderate	90,060
Screen for retinopathy	Type 2 diabetes mellitus	Moderate	67,710
Smoking cessation within past year	Smoking habit	Moderate	711,392
Vaccination for hepatitis B	Hepatitis C	Moderate	14,534
ACEI/ARA2 treatment	Heart failure	High	11,394
Beta blockers treatment	Heart failure/Ischemic heart disease	High	69,155
Antiplatelet/anticoagulant treatment	Atrial fibrillation	High	16,260
Antiplatelet treatment	Cerebrovascular accident/Ischemic heart disease	High	12,916

*Hb* haemoglobin, *LDL* low-density lipoprotein, *ACEI* angiotensin-converting enzyme inhibitor, *ARB* angiotensin II receptor blocker

Health professionals can also choose to view the reminders in their daily schedule of patients. The reminder provides a link to the patient’s electronic health record, from which the pertinent action can be taken (generate a prescription, record a vaccination, request a laboratory test, or record a clinical variable). There is also an option to ignore or exclude a recommendation for a particular patient and enter a comment about the reason for the exclusion.

#### Randomization

**Fig. 3:**
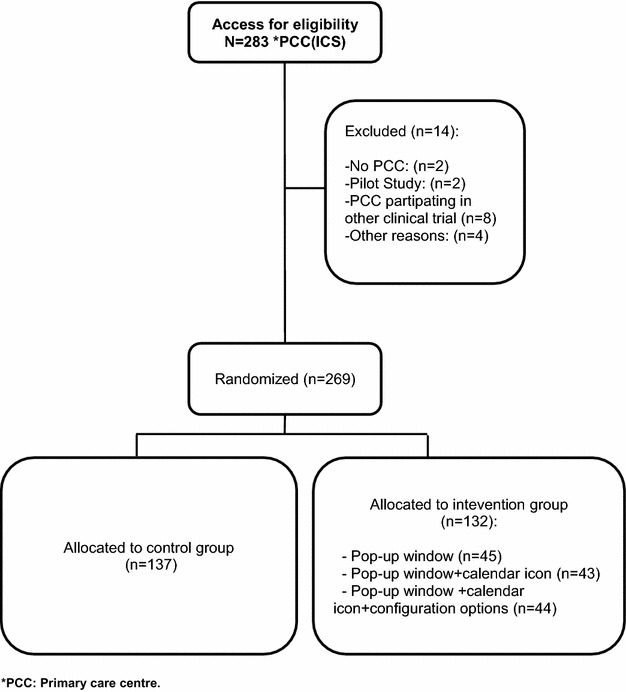
Algorithm for the randomization to each study group

The PCC will be the unit of analysis, so that all professionals from the same centre will be assigned to the same arm of the trial. Randomization will be done with the Stata/SE (v. 11.2, StataCorp) module for the design of randomized clinical trials (Ryan 1990). With the goal of achieving a balanced distribution of the quality of care by PCC in each arm, centres will be stratified by the quintiles of EQA results in the month prior to study initiation. The procedure will consist of obtaining the ICS list of PCCs, importing the data in STAT format, applying the inclusion and exclusion criteria, and adding the EQA value for each PCC. The PCCs will be allocated in randomized blocks of 6, 12, and 18 to achieve the same number of PCCs in each of the study groups (Fig. 3).

#### Intervention groups

The reminders can appear in the electronic health record screens in 3 levels. Each intervention group will receive a distinct, incremental presentation of the reminders: pop-up only, pop-up plus calendar icon, and pop-up plus calendar icon, with a customizable option.

**Fig. 4:**
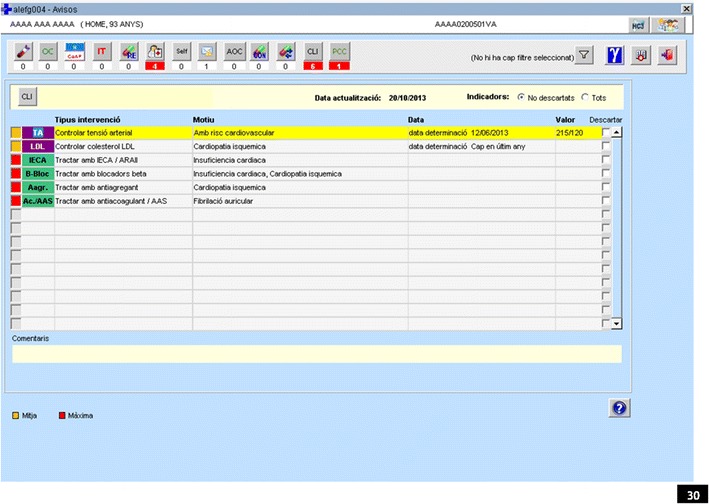
Detail of the one type of intervention screen (pop-up window) to which health professionals have access

1. Pop-up window
  Health professionals allocated to this group will access a patient’s electronic health record; if a reminder has been generated for that patient, a pop-up window will present all of the patient reminders. No other display option will be available. The pop-up appears automatically to the professional on accessing the patient record from their calendar list of patients with an appointment. Before entering any other information they have to close the pop-up or click on the reminder to address the issue.
2. Pop-up + calendar icon
  Each health professional has an online calendar with the list of patients scheduled for the day. The professionals allocated to this arm will be alerted to any reminders pertaining to each patient on the daily schedule. A square orange or red icon will appear next to the patient’s name, indicating the most important reminder (moderate or high) generated, with a small number showing the total number of reminders for that patient. This icon will be linked to the patient’s electronic health record with the pop-up window described for the first study arm.
  This level of intervention (in addition to just the pop-up window) gets a calendar icon. As for the pop-up, health professionals sees this window every time they access the patient’s record from the appointments calendar, the content of the pop-up being the unaddressed reminders, As for the calendar icon, the professionals see them every time they navigate to the calendar list of patients with an appointment.
3. Configurability (pop-up + calendar icon + configuration options)
  The default configuration will show all reminders generated for a specific patient. Health professionals allocated to this group will be able to modify the default configuration, adding filters to select only the reminders they want to see integrated into the electronic health record. This option will be added to the pop-up window (which will still allow access to the expanded list of reminders) and the calendar alerts (which will continue to indicate the total number of reminders generated). Screenshots and detailed description of the intervention (Fig. 4).

#### Length of the intervention

The trial is designed to last one year. After 6 months, the main outcome variable will be analysed and the study will be halted if there is more than 10 % difference between the control group and any of the intervention groups.

#### Training

Intervention groups will receive the usual ICS training about the functionality of the electronic health record system, with some changes. The usual training is provided for regional ECAP coordinators, who then train the health care teams in the region. For the purposes of the clinical trial, training will be provided only for teams allocated to intervention groups. The directors of each of these PCCs will be contacted before the training process begins.

One 2-h session will be provided for each of the three intervention groups during the first 3 weeks of the trial, to be attended by the regional ECAP coordinators and PCC directors, representing their teams. The research team will explain to each group how the reminder system works in that specific arm of the study. The PCC directors will then be responsible for training the rest of the professional team, independently and without supervision or follow-up by the research team, who will nonetheless be available to answer questions that may arise.

### Pilot study

Before the intervention begins, a 2-month pilot study will be conducted in two primary care teams under actual working conditions (patient situations and work setting). All professionals on these two teams will have access to all three reminder formats. In this phase, any problems will be addressed. At the end of the pilot study, an ad hoc survey will be administered to assess the participant’s’ response to the intervention, their level of satisfaction, and any additional suggestions for improving the reminder system. Throughout the intervention, the teams participating in the pilot test will continue to receive the reminders in the format of their choice but will be excluded from the analysis of the clinical trial outcomes.

### Outcomes

The main outcome variable is the health professional’s adherence to the reminder, measured as the proportion of patients receiving the recommended attention (yes/no) and the time elapsed between the first reminder message and implementation of the recommended action (months). Reminders will be considered “resolved” when the professional has completed the recommended actions (Table 2).

**Table 2:** Reminders seen by the health professional, corresponding clinical condition, and the resolution

Reminder	Clinical condition	Resolution
Control blood pressure	Arterial hypertension/Elevated cardiovascular risk	Most recent blood pressure reading within 1 year shows ≤140/90 mmHg or 150/95 mmHg, depending on cardiovascular risk status
Control glycated hemoglobin	Type 2 diabetes mellitus	Most recent glycaemia test within 1 year shows HbA1c ≤ 8 %
Control cholesterol	Cerebrovascular accident/ischemic heart disease	Most recent cholesterol test within 1 year shows LDL ≤120 mg/dl
Screen for retinopathy	Type 2 diabetes mellitus	Record of thorough eye examination within past 2 years
Encourage smoking cessation	Smoking habit	Last record says “non-smoker”
Vaccinate for hepatitis B	Hepatitis C	Record of vaccination
Treat with ACEI	Heart failure	Active ACEI/ARB prescription
Treat with beta blockers	Heart failure/Ischemic heart disease	Active beta blockers prescription
Treat with antiplatelet or anticoagulant drugs	Atrial fibrillation	Active prescription for antiplatelet or anticoagulant drugs, as appropriate
Treat with antiplatelet drugs	Cerebrovascular accident/Ischemic heart disease	Active antiplatelet prescription

*Hb* haemoglobin, *LDL* low-density lipoprotein, *ACEI* angiotensin-converting enzyme inhibitor, *ARB* angiotensin receptor blocker, *HbA1c* glycated haemoglobin

### Data collection and management

The ICS primary care information system (SISAP) will be used to identify patients with a health status that could be improved by the health reminders, and execute a weekly update of patient status. The variables required to execute these algorithms will be obtained from the clinical registry of electronic health records. They include patient diagnoses (by CIE-10 code), primary care visits, clinical variables, drug prescriptions, and vaccinations. In addition to the variables specific to the intervention (resolution of the reminder), other explanatory and descriptive variables will be collected (Table 3). Data for the EQR (quality of data recording) will be collected from the SIDIAP database (*Sistema d’Informació per al Desenvolupament de la Investigació en Atenció Primària*) (Bolíbar et al. 2012). Moreover, the Medea socioeconomic index (Domínguez-Berjón et al. 2008). The EQA, EQPF (prescription quality standard), and other variables at the level of the professional or PCC will be obtained from the ICS information system.

**Table 3:** Variables included in multi-level analysis

Variable	Explanation
Variables at the reminder level	
Type of clinical activity required^a^	Preventive or follow-up activities, immunizations, treatment initiation
Reminder generated	Yes or no
Clinical situation can be improved	Described in Table 1
Age and sex^a^	Older populations generate a higher number of clinical situations that can be improved because of the relationship between age and morbidities
Comorbidity, weighted according to CRG^a^	CRG (Clinical related groups) classify patients according to morbidity, based on diagnoses, status and severity in different categories. Each category has a morbidity weight. A priori, greater morbidity should generate a greater number of improvable clinical situations
Variables at the professional/PCC level	
Socioeconomic level of the PCC	Medea Index
Rurality	Construct based on size of municipality and population/Km^2^
Teaches family medicine	PCC or team member is accredited for postbaccalaureate training in Family and Community Medicine
Teaches basic nursing	PCC or team member is accredited for baccalaureate training in Nursing
Percentage of assigned population visiting the PCC previous year	Percentage of the assigned population who visited the PCC/individual health professional during the year prior to the study. A priori, professionals who see a larger percentage of their assigned patients have more opportunities for interventions to improve clinical situations.
Frequency of patient visits	Number of visits per year/population with at least one visit in the previous year. A priori, a greater number of patient contacts with a health professional will yield more opportunities to correct clinical situations.
EQA	Synthetic clinical indicator consisting of a group of indicators based on scientific evidence.
EQPF	Synthetic indicator developed by the ICS Department of Medication Strategy, including 21 indicators related to the quality of the prescription
EQR	Synthetic indicator that analyses the difference between population prevalence and a patient cohort, which identifies professionals with a good level of recording quality

*EQA* quality care indicator, *EQPF* prescription quality indicator, *EQR* quality of data recording, *CRG* clinical related groups, *PCC* primary care centre

^a^Anonymized data from patient records

### Sample size

A 10 % difference between the proportion of resolved reminders and the assumed proportion in the intervention group of 59 % will be considered clinically relevant (Demakis et al. 2000). Assuming an alpha error of 0.01, 95 % power, and 10 % loss to follow-up, and correcting for an intraclass correlation in the PCCs of 0.05, each of the 4 study groups will require 15,950 reminders, a total of 63,800. The estimated number of reminders to be generated is shown in Table 1. Nonetheless, due to the design of the computerized tool, the need for cluster randomization, and the availability of the universal sample, it is not considered necessary to limit the study to the calculated sample size.

### Statistical analysis

Due to the correlation between the reminders for the same patient, the patients assigned to the same professional, and the professionals at the same centre, hierarchical modelling will be used, allowing simultaneous estimation of a factor effect at various levels.

### Resolution of the reminder

To estimate the effect of the intervention on improving the resolution of each reminder, a two-level analytical strategy will be used:

- Professional/PCC level
  The effect of the quality variables—EQA, EQR, and EQPF—on the resolution of the reminder will be evaluated at the aggregate level: percentage of reminders resolved. The Pearson correlation coefficient and scatter plots will be used for this purpose.
- Reminder level
  Hierarchical or multilevel methods will be used to take into account the structure of the information and be able to introduce variables from different levels as adjustment variables in estimating the intervention effect. Multilevel logistic regression models will be fitted. The analytical strategy will be to estimate the intervention effect, taking into consideration the randomized professionals; next, the effect size will be estimated by introducing the quality variables from the professional level (EQPF, EQA, and EQR). Finally, we will determine whether introducing variables from the reminder level changes the intervention effect. A subgroup analysis will also be done, by type of reminder.

### Time to resolution of the improvable clinical situation

Time to resolution will be estimated by the Kaplan–Meier method and comparisons will be done using the log-rank test. Multilevel survival analysis will be used to homogenize the data structure (Yau 2001). The analytical strategy is the same as described above: an initial model will be adjusted to estimate the intervention effect, introduce the professional-level variables (EQA, EQR, EQPF), and then determine whether the variables at the reminder level change the estimated intervention effect.

The level of significance will be established at 5 %. All analysis will be done using the Stata/SE software, version 11.2 (StataCorp 2012).

### Research ethics

The study design followed ethical guidelines contained in national legislation and the international standards defined in the Declaration of Helsinki and Tokyo. The study protocol was approved by the Committee on Clinical Research Ethics of the Jordi Gol Institute on Research in Primary Care (*Institut Universitàri d’Investigació en Atenció Primària*, IDIAP Jordi Gol).

All data extracted from the electronic health records system to create the reminders, as well as all data collected during the study for later analysis, will be anonymized so that it will be impossible for the research team to identify individual patients of the participating health professionals. A computer algorithm will be used that shows patient identification only to the primary care professionals (physicians and nurses) responsible for the patient’s care.

Patients are only indirectly affected by the trial because the researchers are not involved in any intervention or interaction with patients, all patient data are anonymized in accordance with national data privacy laws and National Health System policy, and the intervention is based entirely on best practice recommendations drawn from existing scientific evidence, published in clinical practice guidelines, and included in the objectives of the Catalan government’s Health Plan (*Pla de Salut*). Indeed, if the intervention results in better adherence by health professionals in the ICS primary care network to these evidenced-based recommendations, patients will benefit from the outcomes achieved by this clinical trial.

### Study oversight

There isn’t an independent data monitoring or trial steering committee.

## Discussion

The results of the present clinical trial will contribute new evidence on the impact of computerized point-of-care reminders on the adherence to clinical recommendations by primary care professionals, using an established feedback system. The study will involve almost all health professionals in the ICS primary care network and the population they serve, and therefore will be highly representative of clinical practice in the primary care setting. If the intervention is shown to be effective, the improvements should be almost immediately apparent, benefiting almost 5 million patients in the National Health System in Catalonia. Since the interventions will involve a high number of patients, small improvements in effectiveness could have a great impact on population health, according to the literature, both in terms of direct benefit and side effects. For example, increased use of antiplatelet therapy in patients with cerebrovascular disease reduces the number of coronary or cerebrovascular episodes and the associated mortality, but the number of haemorrhages also increases as a consequence of the treatment. To avoid undesirable effects to the extent possible, we selected indicators for which risk–benefit analysis has provided evidence that clearly favours the intervention.

Previously published studies have described better effectiveness when the feedback is a response to low adherence to clinical practice guidelines (Jamtvedt et al. 2003). In our study population, there is no low level of adherence to recommendations concerning the conditions we are studying, because health professionals have already been receiving performance feedback and lists of patients for 5 years during which their indicators have raised significantly. On the other hand, in contrast to many of the published studies, our design adds the reminders to an established feedback system that will be used in all arms of the study; this may lead to a smaller effect size. Holt et al. compared the use of reminders to a control group with no supporting tools (Holt et al. 2012). Therefore, we selected a 10 % intervention effect as clinically relevant.

Our study has some limitations. Some authors have suggested that “pay for performance” incentives may lead to better recording rather than better clinical practice (Bell and Levinson 2007; Petersen et al. 2006). A Cochrane review concluded that despite the increasing growth in this type of payment system, there is insufficient evidence for or against any impact on improving primary health care services (Scott et al. 2011). The compensation for objectives related to the indicators included in the intervention is low, not exceeding 750 euros per physician and 380 euros per nurse. In any case, this effect would occur in both the control group and the intervention groups; therefore, it would be unlikely to have an impact on the results that may be obtained. On the other hand, most of the selected indicators would not be susceptible to manipulation because they are based on laboratory results such as LDL cholesterol levels or HbA1c levels in patients with diabetes, or prescription data for specific drugs (e.g., antiplatelets, anticoagulants, beta-blockers, angiotensin-converting enzyme inhibitors). Therefore, it is likely that any recording changes would have real impact on health status.

Studies based on data from electronic health records can also be limited by the quality of the record, and any clinical records software such as ECAP could have a certain level of under-recording. Nonetheless, this limitation should affect both the intervention and control groups, and most of the reminders are related to health problems that are long-term indicators with a prevalence similar to what has been reported in the literature. This leads us to conclude that potential under-recording is not a major problem of the study design.

Finally, the health professionals participating in our study cannot be blinded to the intervention, which could possibly generate an information bias. Nonetheless, we selected cluster randomization because most studies of this type use this design; we decided against any design in which the same health professional would have some patient records with reminders and others without. In a study by Chambers et al. participants in one study arm received sporadic reminders about flu vaccine, others received them consistently, and a third group received no reminders (Chambers et al. 1991). The outcomes were worse for the sporadic reminders than for the control group, suggesting the hypothesis that health professionals become dependent on the reminder: if it does not appear, they may assume that the patient does not need the vaccine, which could result in a harm rather than a benefit to the patient.

## Abbreviations

CEIC: Clinical Research Ethics Committee (*Comité Étic d’Investigació Clínica*)
ECAP: electronic health record system of the Institut Català de la Salut in primary care (*Estació Clínica d’Atenció Primària*)
EHR: electronic health record
EQA: quality care indicator
EQPF: prescription quality indicator
EQR: quality of data recording
GP: general practitioner
ICD: International Classification of Diseases
ICS: Catalan Institute of Health (*Institut Català de la Salut*)
IDIAP: University Research Institute on Primary Care (*Institut Universitari d’Investigació en Atenció Primària*)
LDL: low density lipids
PCC: primary care centre
SIDIAP: information system for research on primary care (*Sistema de Informació pel Desenvolupament d’Investigació en Atenció Primària*)
SISAP: primary care database of the ICS (*Sistema d’Informació dels Serveis d’Atenció Primària*)
HBP: high blood pressure
CVA: cerebrovascular accident
IC: ischemic cardiomyopathy
FA: atrial fibrillation
ACE: angiotensin-converting enzyme inhibitors
ARB2: angiotensin receptor blockers
CRG: clinical related groups
